# Emotional interdependence: the key to studying extrinsic emotion regulation

**DOI:** 10.1186/s41155-022-00237-9

**Published:** 2022-11-04

**Authors:** Ana Kinkead, Christian Salas Riquelme

**Affiliations:** 1 Universidad Autónoma de Chile, Av. Pedro de Valdivia 425, Providencia, Santiago, Chile; 2 Faculty of Psychology, Universidad Diego Portales, Santiago, Chile

**Keywords:** Extrinsic emotion regulation, Emotional interdependence, Recursiveness, Romantic dyad, Couple satisfaction, Shared reality

## Abstract

The literature on *extrinsic emotion regulation* or the intention to modify other people’s emotions has grown in recent years, accompanied by proposals in which its definition is made more precise, the way to understand it in relation to other related processes is delimited, and the consequences of its use in the quality of close relationships are evidenced. Conceptual reviews on this topic recognize the importance of examining the affect and dyadic dynamics that arise between those who regulate each other extrinsically. This dynamic refers to *emotional interdependence*, the potential of the members of a dyad to shape each other’s emotions reciprocally, particularly in those who share a close bond, such as that of a romantic couple. There is little theoretical development regarding the relevance of this characteristic in relation to EER. This article has two objectives: (1) to make a narrative synthesis of the characteristics that define EER and (2) to expand and complexify the existing model by including the *emotional interdependence* as a vital component in the understanding of the functioning of EER. Lastly, the role of emotional interdependence in the emergence, maintenance, and satisfaction concerning couple relationships is made explicit through phenomena such as shared reality.

## Introduction

Emotion regulation refers to a process by which humans voluntarily manipulate the duration, intensity, and type of emotions they experience (Ray-Yol & Altan-Atalay, 2020). Studies on this process have shown that people can use both intrapersonal and interpersonal strategies to achieve this goal. Regarding intrapersonal strategies, it has been described that they seek to influence one’s own affective sphere by manipulating when and how emotions are experienced (Gross & Thompson, 2007). With respect to *interpersonal strategies*, these are usually grouped according to the course of the interactions, i.e., whether people use others to regulate their own emotions (intrinsic interpersonal emotion regulation) (Messina et al., 2021; Zaki & Williams, 2013) or seek to modify the emotional trajectory of another person (extrinsic interpersonal emotion regulation). *Interpersonal emotion regulation* (Niven, 2017; Zaki & Williams, 2013) is also known as social regulation of emotions (Reeck et al., 2016).

Historically, the study of emotion regulation has focused on the regulation of one’s own emotions (intrapersonal level). In contrast, the regulatory processes—both intrinsic and extrinsic—that occur at the *interpersonal* level have been less studied (Campos et al., 2011; Hofmann, 2014). Regarding *extrinsic emotion regulation* (EER), its study has gained relevance in recent decades due to the evidence of its role as a social support mechanism related to the strengthening of bonds between people (Coo et al., 2020; Debrot et al., 2013) and its relationship with emotional well-being (Williams et al., 2018) and mental health (Christensen et al., 2020; Güney, et al., 2015; Horn & Maercker, 2016). The literature also shows the relationship between EER with unfavorable effects on relationships, for example, with marital problems, more negative and less positive emotional expressions (Becerra et al., 2020; Gottman & Levenson, 1992), and emotional dysregulation (Weber & Herr, 2019).

Interest in EER has also been reflected in the publication of conceptual articles that have attempted to define the core attributes of this process and the elements that differentiate it from other regulatory mechanisms. For example, reviews by Niven (2017) and Zaki and Williams (2013) have pointed out that in order to examine EER, one must attend to the goals pursued by the regulatory action, the (social) context in which it occurs, and the processes underlying it (whether or not the outcome depends on the response of the other person), as well as consider the recognition of the intention of who regulates. However, to date, the discussion has tended to focus on the relationship between the use of extrinsic regulation strategies and personality traits/psychopathology (Christensen et al., 2020; Niven et al., 2012), as well as their link to the situational demands of the person regulating (Chen & Liao, 2021). A limitation of these theoretical proposals is that they have only tangentially considered the dynamic aspects of EER. This problem has been highlighted by Dixon-Gordon et al. (2015), who have pointed out the need to develop innovative paradigms that capture the dyadic and dynamic nature of extrinsic regulatory processes, especially when they happen in individuals who share a close relationship (e.g., parent–child relationships or couples). This limitation of the literature is relevant in that some authors have pointed out thathe dynamic aspects of EER do not only occur in close relationships (Turliuc & Jitaru, 2019), raising the question of how the type of bond (partner, friend, or acquaintance) may influence the pattern of expression and emotional intensity of the response differently (Jones & Barnett, 2020; Lindsey, 2019). In this regard, Niven (2017) has proposed that the relational context (i.e., the nature of a relationship, the duration, and intimacy between those who regulate each other) and the dynamic nature of emotional processes (their change over time and/or their change due to social feedback received) can modify the regulatory strategies they use with different consequences for those interacting.

**Fig. 1:**
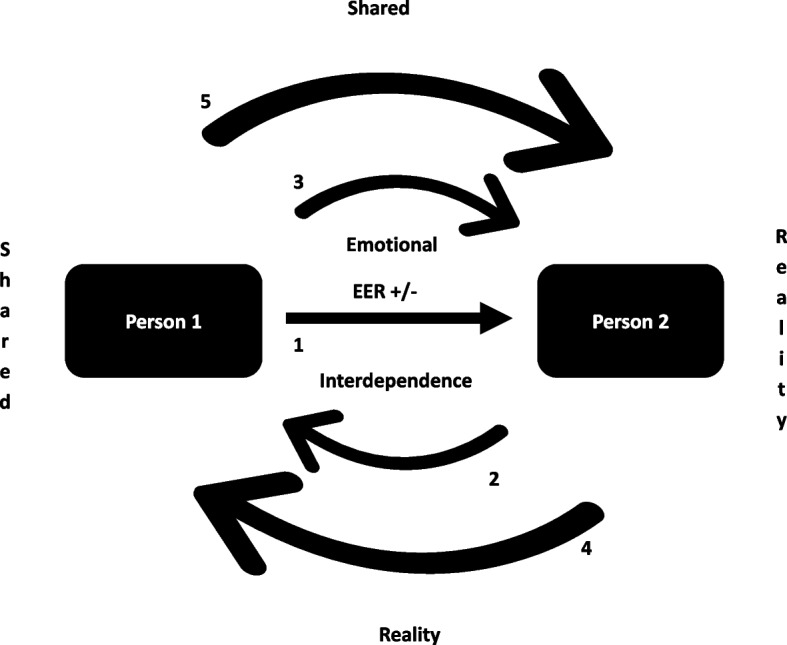
Emotional interdependence: the key to studying extrinsic emotion regulation in romantic dyads. (1) Positive or negative EER of person 1, generates a positive or negative emotional response in person 2. (2) This response, in turn, generates positive or negative emotions in person 1, along with other associated phenomena. (3) The couple as a circular system, emotions and other related phenomena continue in a reciprocal and iterative loop, which allows the couple at some point to build meanings around their identity as a dyad and the quality of their relationship, between others. (4) Since the effects of the EER occur within the framework of emotional interdependence, the identity of the dyad allows more complex phenomena such as a reality that is mutually shared (5), which in turn will be reinforced according to the successive emotional responses that continue to occur among its members

This theoretical positioning paper supposes the evaluation of progress in a specified area, for which it pursues the following objectives: (1) to make a narrative synthesis of the postulates or characteristics that define EER and (2) to expand and complexify the existing model by including the phenomenon of *emotional interdependence*—the potential of the members of a dyad to shape each other’s emotions reciprocally—as a central component in the understanding of the functioning of EER. To achieve these objectives, we will consider the dyadic interaction of romantic partners as the main study object, as this is where emotional interdependence is expressed most clearly (Schoebi & Randall, 2015). The article will be organized by first presenting the three defining characteristics of EER according to existing reviews, and then, the phenomenon of interdependence and its implications will be included (Fig. 1).

## Defining characteristics of EER

### Characteristic 1: The target of EER is other people’s emotions

One definition of EER on which there seems to be some consensus is its conceptualization as “an action performed with the goal of influencing another person’s emotion trajectory; it can aim to decrease or increase either negative emotion or positive emotion” (Nozaki & Mikolajczak, 2020, p. 3). These characteristic positions the emotional dynamics outside the individual, in the person who is regulated, or the object of regulation. Initially, this characteristic was referred to by Rimé (2007) as interpersonal *emotion regulation*, describing the social exchange of emotional experiences between people after experiencing affectively intense moments. Later, Niven et al. (2009) used the term *interpersonal affect regulation* to account for the emotional modification of others using regulatory strategies.

It is relevant to note that the study of EER strategies has been part of the literature on *social support* and *coping* (Dixon-Gordon et al., 2015) or *prosocial behavior* (Nozaki & Mikolajczak, 2020). However, EER can be distinguished from these other processes by its more immediate effects and its exclusive focus on the intention to increase, decrease, or maintain both negative and positive emotions of other people. On the other hand, the literature on social support, coping, and prosocial behavior has focused on the relationship of these strategies to long-term stress reduction, emotional well-being, and their link to instrumental support.

There are a substantial number of studies that have explored the individual differences in the extrinsic regulatory agent. For example, it has been described that those with high levels of cognitive empathy—the ability to imagine how others feel—and emotional competence—a form of emotional intelligence—tend to employ strategies that reduce distress in others more effectively, use humor as a predominant strategy (Levy-Gigi et al., 2017; Williams & Emich, 2014) and are more likely to attempt to regulate the sadness of ostracized individuals (Nozaki, 2015). Strategic emotion regulation ability, understood as the ability to identify flexible and effective responses, has been related to lower levels of interpersonal conflict (Lopes et al., 2011). It is interesting to note that, although this characteristic of EER considers the interaction between two agents, the literature tends to focus on the experience and characteristics of the regulator, leaving aside the impact that the experience or response of those being regulated may have on the regulator.

### Characteristic 2: EER is an intentional, controlled, and conscious process

Another characteristic of EER is its deliberate or intentional nature (Messina et al., 2021; Nozaki & Mikolajczak, 2020). This feature differentiates EER from other nonvoluntary, nonconscious regulatory processes such as *emotional contagion* or the tendency to experience the emotions of others due to unconscious and automatic imitation mechanisms (Hatfield et al., 1994). Emotional contagion can happen without the conscious experience of the target of EER by the regulatory agent, even if their emotion does influence the emotion of others as a result (Nozaki & Mikolajczak, 2020). EER also differs from other forms of emotional influence, such as *interpersonal modulation*, where the mere presence of others can attenuate negative emotions in the face of external stressors (Beckes & Coan, 2011).

The intentional, controlled, and conscious nature of EER implies a goal-directed process where people guide their behavior by seeking to achieve a higher-order goal (Cloonan, 2019) and deliberately choosing some strategies over others to that end. The literature suggests that the use of EER may be motivated by reasons of reciprocation, commitment, or obligation to oneself or others (Cloonan, 2019), hedonic and cooperative (Cohen & Arbel, 2020), selfish or instrumental (Netzer et al., 2015), altruistic (López-Pérez et al., 2017), or antisocial motivations (Zaki & Williams, 2013). We also know that regulating extrinsically brings costs and benefits for the regulator, and according to the findings of Netzer et al. (2015), people would be aware of this when choosing extrinsic strategies.

Some authors have pointed out the importance of considering the role of non-intentional/deliberate processes in EER in order to complexify the model. For example, it is known that there are nonverbal behaviors implicit in a dyadic interaction that can predict the deterioration of the quality of a relationship; in other words, relationship success is highly dependent on how people spontaneously behave in their relationship (Faure et al., 2018). The above emphasizes the existing discussion regarding the need to think of ER as “a continuum of emotion regulation possibilities that range from explicit, conscious, effortful, and controlled regulation to implicit, unconscious, effortless, and automatic regulation” (Gross, 2013, p. 360). This aspect of the model is especially relevant for the study of clinical disorders, which have been characterized as disconnections between a person’s emotional experience and its conscious interpretation (Aldao, 2013). Consequently, the need to explore the interaction between the voluntary and nonvoluntary aspects of EER and how each of these processes may be affected in specific psychopathologies arises.

### Characteristic 3: EER seeks to increase and decrease both negative and positive emotions

Much of the research on intrapersonal emotion regulation is based on the notion that people strive to feel good and avoid feeling bad. However, this assumption does not seem to hold in all cases (Zaki, 2020). The same is true for EER, which is not exclusively limited to increasing the well-being and minimizing the discomfort of others, as people may also have goals contrary to this in their daily lives (Cohen & Arbel, 2020). At this point, it is important to clarify that emotions are not intrinsically positive or negative; since their valence will ultimately depend on the adaptive function, they have at a given moment (Ekman, 1992). However, when studying basic emotions, the dimension to which they belong is considered; that is, they are conceived along a continuum. On the one hand, there would be the pleasant emotions or those that seek well-being (positive emotions) and, on the other, the unpleasant emotions that are associated with discomfort (negative emotions) (Lang et al. 2008). For the purposes of this work, the dichotomous classification of positive emotions and negative emotions will be used to refer to pleasant and unpleasant emotions, respectively. In this regard, Niven (2015) has pointed out that the emotion-related goals of regulation attempts (generating positive or negative emotions) are influenced by the motivation that underlies them. Prosocial or hedonic motivations may be related to attempts to improve others’ emotions, whereas instrumental or contra-hedonic motivations may generate behaviors that seek to worsen or maintain the way others feel (Niven, 2015; Riediger et al., 2009). In other words, EER is not reduced to the generation of positive emotions but can take different forms depending on its usefulness in each context. For example, when the motivation for EER is to generate pain or discomfort, there is often an implicit belief that fear, anger, anguish, or guilt will bring long-term benefits (Zaki, 2020). In short, EER also has a role in maximizing personal instrumental gains or benefits, even when doing so entails immediate costs or harm to the other person (Netzer et al., 2015).

There is literature that has focused on exploring the existing relationship between positive and negative EER strategies and relationship quality variables and the role of individual and contextual differences in their use. An emotion-regulating strategy is any activity or behavior that deliberately influences affect (Parkinson & Totterdell, 1999). A positive EER implies the intention to make another person feel good through certain behaviors such as making them laugh, while in a negative EER, the behavior is focused on making them feel bad, for example, by criticizing them. In attention to this, it has been reported that there is a close link between the EER of positive emotions and the value attributed to the relationship (friendship), and that this link does not seem to depend on efforts to decrease negative emotions in the other (Chesney, 2018). In romantic partners, positive and negative EER strategies have been associated respectively with levels of couple satisfaction (Jitaru, 2020; Kinkead et al., 2021). Regarding individual and contextual differences, it has been proposed that these could moderate and/or mediate the effects of positive and negative EER. For example, studies by Marigold et al., (2007, 2014) showed that, depending on a person’s self-esteem, making another person feel good could have negative effects on that person. Similarly, the study by López-Pérez et al. (2017) showed that, depending on the context, making a person feel bad can improve that person’s well-being, albeit in the long term. Since positive and rewarding interactions seem to contribute to the quality of marital relationships and the exchange of negative behaviors diminishes it (Jelic et al., 2014), it is relevant to understand the mechanisms by which a negative regulatory interaction generates a positive effect.

### Characteristic 4: EER through emotional interdependence has implications for dyadic functioning

#### Emotional interdependence

A fourth characteristic of EER, often neglected by the literature, is *emotional interdependence*. The notion of *interdependence* originates from the interdependence theory first proposed by John Thibaut and Harold Kelley in the 1950s (Kelley et al., 2003). This theory proposes that interpersonal relationships are defined through interaction processes where each actor influences the other’s experience (Van Lange & Balliet, 2014). This phenomenon describes the strength and quality of *the effects that interacting people exert* on the preferences, motives, emotions, and behavior of others (Rusbult et al., 2002). It should be noted that interdependence does not only refer to the *mutual* (direct) *influence* that the members of a dyad exert on each other but also considers the combination of decisions or attributes exhibited by each member (Whickham & Kenee, 2012). In the affective sphere, *emotional interdependence* has been defined as coordinated patterns of emotional experience and expression that arise in awfully close relationships (Butler, 2011; Butler & Randall, 2013), allowing *the emotions of the members of a dyad to become aligned* over time (Kelley et al., 1983 in Sels et al., 2019). Other authors define emotional interdependence as a temporal interpersonal emotion system that occurs in the context of a social interaction, where the subcomponents of emotion—experience, behavior, and physiology—interact not only within a person but also between people (Butler & Randall, 2013).

Although interdependence is sometimes described as a single dimension, it is better understood as a multidimensional construct. In this regard, and based on the interdependence theory, Columbus and Molho (2021) describe three dimensions: mutual dependence (the degree to which both individuals mutually control each other’s behaviors), conflict of interests versus correspondence (the degree to which one individual’s gain is another individual’s loss), and relative power (the degree to which one individual has greater control over their own and the other’s behaviors than vice versa).

When analyzing dyadic phenomena, interdependence enables an interpersonal analysis of relationships based on four key elements that it shapes (Rusbult et al., 2002): (1) *daily interactions* (the patterns of interdependence describe the opportunities and limitations that characterize an interaction, defining its potential for sympathy, conflict, and exploitation); (2) *mental events* (cognition and affect reflect attempts to understand the meaning of interdependence situations, to identify the appropriate action to take in such situations); (3) *relationships* (the characteristics of interdependence describe the opportunities and limitations that, in turn, characterize the relationships, defining the possibilities of commitment, trust, power, and conflict; and (4) *the self* (people develop relatively stable preferences, motives, and behavioral tendencies as a consequence of adapting to the interdependence situations with which they coexist daily).

#### Interdependence, expression, and emotional exchange

Couples have been a particularly relevant group in the study of emotional interdependence. Several authors have described that the partner becomes an essential regulatory resource in adulthood (Butler & Randall, 2013; Sels et al., 2016; Zaki, 2020); therefore, EER is more frequent in this type of relationship than in work or friendship ones (Martinez-Inigo et al., 2013; Turliuc & Jitaru, 2019).

A distinctive characteristic of couples is the relevance of emotional expression and communication, as they allow emotions to fulfill their function of coordinating social interaction (Anderson et al., 2003). For example, the emotional expression behind verbal and nonverbal courtship behaviors (sustained eye contact, loud laughter, emphatic head nodding, and exchanging affectionate gestures such as caressing) serves to attract and maintain the attention of a potential partner (Moore, 2010). Reports on emotional expression have also been obtained by studying voice modulation during a conversation, showing that couples tend to show greater connectedness and intimacy than acquaintances (Farley et al., 2013). Other researchers affirm that the closeness that exists in couples not only facilitates the perception of emotions in others, especially when it comes to negative emotions (Clark et al., 2002), but also allows for greater intensity and frequency of emotional expression in them (Kane et al., 2018). In summary, the use of the construct of emotional interdependence favors the observation or recording of extrinsic regulatory processes in romantic dyads (Lindsey, 2019). For example, Coan et al. (2017) and Morris et al. (2018) show that neural activity during the anticipation of threat decreases significantly when the source of extrinsic regulation is the partner, compared to when it is an acquaintance or friend. Moreover, Liu et al. (2021), in addition to noting a greater stress-alleviating effect when the regulator is the partner, identified that the quality of the couple and partner’s commitment could modulate this result, making the participants feel more confident.

In sum, the fact that members of a romantic dyad can show or express their emotions more fluently, as well as to perceive the emotions of their counterpart more easily, opens a way to understand the role of interdependence and the effects of EER both on each of its members and the dyad (Martinez-Inigo et al., 2013; Niven et al., 2015). An example of how EER would occur under the condition of emotional interdependence would be *capitalization*: an interpersonal form of regulation strategy in which positive events or news are shared with close ones (Gable & Reis, 2010). Studies on couples report that when one partner responds actively and constructively (showing enthusiasm rather than a passive or destructive response), relationship benefits are observed around satisfaction, intimacy, commitment, trust, and feelings of closeness and security (Gable & Reis, 2010; Gable et al., 2004), as well as gratitude (Woods et al., 2015). For its part, the work by Caldwell et al. (2019) examined how intrusive rumination (a regulatory strategy described as repetitive, passive, and focused cognition about one’s own causes and consequences of emotional distress) from both members of a relationship contributed to couples’ conflict. The authors reported that when self-regulatory capacity improves in one of the actors, rumination in that actor attenuates, and, eventually, couples’ conflict decreases. The work by Horn et al. (2018) also accounts for the extrinsic regulatory effect in interdependent relationships. The authors explored the intra- and interpersonal effects of positive humor on emotional changes in romantic couples in the context of everyday life, which would be mediated by an increase in psychological intimacy. Their findings indicate that daily positive humor in one of the dyad members serves as an EER strategy that affects both members of the couple, considering feelings of psychological intimacy as an indirect socio-affective mechanism.

#### The role of interdependence on the effects of EER in couple relationships

Some authors have come to consider emotional interdependence as the cornerstone of couple relationships (Sels et al., 2019; Whickham & Kenee, 2012). This is possible because, in the case of a couple, we would be facing a relational system characterized by the circularity and recurrence of their interaction, so every action performed within this system can be understood as a reaction and vice versa; every reaction becomes the cause of subsequent behaviors and actions (Campos & Linares, 2002 in Moreno-Manso et al., 2015).

It is important to distinguish emotional interdependence from another similar phenomenon that has been widely studied: emotional coregulation. Emotional coregulation has been defined by Butler and Randall (2013) as those processes that, in a relationship between peers or members of a dyadic emotional system, are carried out through an oscillating pattern of affective arousal and dampening of negative emotions that dynamically maintains an optimal emotional state for the couple. In other words, a mutual influence can be observed, but its goal is to dampen negative emotions and restore homeostasis lost during an interaction. Although coregulation also adopts the dyad as the unit of analysis and considers interpersonal regulatory processes, it is distinguished from EER because the former is an unconscious or involuntary process (Butner et al., 2007; Fraley & Shaver, 2000) that is necessarily established once distress alleviation is achieved (Butler & Randall, 2013).

The consequences of making one’s partner feel good or bad influence the creation of other functional phenomena in a romantic dyad. To regulate extrinsically, in addition to the identification of the other person’s emotional state, processes of social cognition (the processes underlying social perception, engagement, and interaction) and affective and cognitive empathy must be activated (Hallam et al., 2014). Therefore, close relationships afford us opportunities to create and maintain meaning systems as *shared perceptions* of ourselves and the world (Andersen & Przybylinski, 2017). These shared perceptions are fundamental in creating and maintaining lasting social bonds as they strengthen *shared* beliefs about how the world works inside and outside the relationship (Murray et al., 2018). Consequently, extrinsically regulating a partner’s emotions would also involve taking another person’s perspective and acknowledging that there is another viewpoint, which can either challenge the concept of *shared reality* or preserve it by helping to explain how aspects of the world may be perceived differently by two different individuals (Hodges et al., 2018). Shared reality is a central aspect of interpersonal relationships that refers to the subjective experience of sharing a set of inner states (e.g., thoughts, feelings, or beliefs) with another person in a way that, in addition to helping to verify one’s own conceptions of oneself or to align oneself with the other person’s viewpoints, induces the co-creation of shared meaning (Andersen & Przybylinski, 2017). According to Rossignac-Milon and Higgins (2018), people construct this shared reality to achieve closeness and intimacy—relational motives—and to make sense of the world—epistemic motives. For example, when facing an unexpected or stressful event, relationships offer a way to restore meaning and order to the world or the relationship (although they can also be a source of disorder and confusion), forcing people to review the shared realities (Murray et al., 2018). Shared reality has been described to progress through four cumulative phases (Rossignac-Milon et al., 2018): (1) relationships are often initiated when people discover shared feelings; (2) this facilitates the co-construction of dyad-specific shared practices; (3) then, partners form an interdependent web of shared coordination; and (4) ultimately, partners develop a shared identity (the risk of relationship dissolution is present at each stage). In each of these phases, it is the sharing of everyday experiences that promotes a merging or alignment in how the world is understood and interpreted (Berger & Kellner, 1964 in Rossignac-Milon & Higgins, 2018), and convergence of attitudes and emotional responses is achieved in the dyad (Anderson et al., 2003; Butler, 2015). When a romantic dyad uses *extrinsic emotion regulation strategies* in its daily life, it generates, on the one hand, a space where the experience of making the partner feel good or bad allows merging or aligning shared meanings (constructing a shared reality), and, on the other hand, that people acquire a relevant role for their counterpart, perceiving each other as “instruments” or means to achieve certain goals (Orehek & Forest, 2016; Rusu et al., 2018). Therefore, it is possible to understand the intimacy bond formed, which is expressed by the search of the partner as a regulating agent in times of stress (Hazan & Shaver, 1987). Since this entire process takes place in a context of interdependence, helping others to improve their emotional state provides the person being regulated with an emotional buffer against negative life events and a sense of efficacy and social value to those who regulate (Salovey et al., 2002).

## Discussion

Each of the characteristics or postulates described above could benefit from an interdependence approach. In the case of the first characteristic or postulate (*the target of EER is other people’s emotions*), the strategies aimed at modifying the partner’s emotions can be adjusted according to the assessment of the emotional state expressed by the interactants (Niven, 2017). For example, it would be possible to examine whether the strategies used to modify the emotions of another person are susceptible to change during the same interaction, considering the recursive effects (interdependence) it has on the couple’s identity (shared reality). In this way, people are more likely to increase their partner’s concern if they see that the partner is taking an apparently fundamental problem, or one that they should be concerned about, too calmly (Zaki, 2020). Considering the concept of interdependence gives a *dynamic character* to the study of EER in couples, making it necessary to examine not only the strategies that may arise at a given moment but also their variation according to the feedback received by their interactants. Regarding the second defining postulate (*EER is an intentional, controlled, and conscious process*), the possible interaction between this characteristic and the level of perception of interdependence that the dyad itself has may have been interesting. Columbus and Molho (2021) state that people have an often-intuitive grasp of their interdependence with others (e.g., recognizing that one’s well-being may be tied with that of another person), and that these perceptions are based on a shared social reality (i.e., they are more accurate). In addition, people who are aware of the mutual dependence in the relationship would show more prosocial behaviors, i.e., actions that benefit the other person. Consequently, this relationship raises research questions regarding whether there are differences in the effects of EER depending on the level of awareness of emotional interdependence or the effects that the interaction would have on the couple. With respect to the third characteristic (*EER seeks to increase and decrease both negative and positive emotions*), considering that a negative EER strategy has reciprocal effects (it affects the actor and not only the one to whom the regulation is directed), the interdependence relationship could be mediating the construction of shared meanings in the dyad and, thus, determine both medium- and long-term beneficial effects for it. This is because some effects expected from the use of positive and negative EER strategies are congruent with the type of strategy employed, but there are also inconclusive or contradictory results for the couple and other types of relationships (López-Pérez et al., 2017; Marigold, et al., 2007). In the first place, if we consider that people need frequent personal interactions, ideally affectively positive and free from conflicts and negative affects (Debrot, 2012), a positive EER would fulfill in part the purposes described, being coherent with favorable and expected results for a couple relationship. Secondly, it is known that negative experiences in relationships have a stronger detrimental effect on cognitive, emotional, and behavioral functioning than positive ones (Palmwoods & Simons, 2021). However, negative emotions (although unpleasant) would serve a communicative function: they would indicate a malfunction or maladjustment in the relationship, potentially motivating people to address their problems or improve the relationship (Baker et al., 2014).

In sum, emotional interdependence—due to its reciprocal and iterative nature—would provide a *recursive link* between EER and its effects on the dyad; in other words, the fact that EER has consequences for couples’ functioning suggests that interdependence would serve a feedback role for the creation of shared realities and other associated phenomena in the dyad. Therefore, including interdependence in the notion of EER in couples implies, in addition to adding a second-order element in the analyses (i.e., another person), incorporating a weakly recognized link such as the existing relational context, which helps to go beyond the immediate effects of EER and think of it as a phenomenon that also allows for the creation of shared meanings.

Given this background, a productive way to look at EER is through emotional interdependence. However, in order to observe a phenomenon that is more dynamic than static, incorporating interdependence into EER poses new challenges at the methodological level. In this sense, the *use of diaries* as one of the most used methods in this field could reflect this change more easily, as it allows examining events and experiences in their natural and spontaneous context, minimizing the amount of time between the experience lived and the respective report. This also implies that the use of diaries in research can take advantage of information that might be overlooked under traditional designs involving cross-sectional assessments (e.g., surveys) or widely spaced longitudinal assessments (Laurenceau & Bolger, 2005). It would be interesting to add the perception of how much one's behavior affects the partner's behavior and the value attributed to this effect on the relationship itself to the research records (own emotional response, stressful events, strategies used, among others) (Rossignac-Milon et al., 2021).

## Conclusion

The use of EER strategies, especially in relationships that form a particular unit, such as couples, produces effects on the functioning of the relationship that are reciprocal among their members. It is in the understanding of this reciprocity where the need to consider *emotional interdependence* in the definition of EER lies. However, conceptual reviews examining EER have not directly or explicitly considered this condition of interdependence and continue to address it from an individual perspective. By not incorporating those who also exert an effect on this phenomenon in the dyad, valuable information for understanding affective dynamics in close relationships is left out. This article aimed to synthesize the central postulates that give life to the notion of EER and to introduce emotional interdependence as a key factor in a phenomenon that cannot be understood from a unidirectional perspective. In terms of new questions, a next step would be to investigate the predictive power of EER in the creation of shared reality in the couple, as well as in the strengthening of the latter, considering the fundamental belongingness need (Baumeister & Leary, 1995). A second step for future studies is that since interdependence theory explains behavior from those attributes that reside in the dyad’s interactions (and not in the individual’s own attributes) (Kelley et al., 2003), another area to examine is related to the perception of the dyad’s own interdependence and how this would be linked to an EER oriented to generate prosocial behaviors (Columbus & Moho, 2021). Along the same lines, in addition to the perception of interdependence, it would be relevant to consider the expectations regarding the role that interdependence plays in the maintenance of close relationships, as people base their decisions mainly on the possible options that best lead to the desired results (Baker et al., 2020). A third step would be to examine the full range of relationship functioning, as employing negative regulation strategies in the dyad might not necessarily lead to negative outcomes. Since negative emotions are not inherently harmful and can serve essential functions in relationships (Baker et al., 2014), it would be novel to characterize those negative extrinsic regulation strategies that have beneficial effects on the dyad and to understand the conditions under which they can lead to a strengthening of the relationship. Lastly, it is expected that this approach to EER will be a step towards incorporating other phenomena of equal importance for the dynamics and quality of a couple relationship, such as dyadic identity (when the members of a relationship perceive themselves as a specific and important part of that relationship) (Acitelli et al., 1999) and changes in the psychological intimacy of a couple as a mechanism by which emotions are regulated in the dyadic context (Horn et al., 2018).

## Data Availability

Not applicable.
