# Correction: Emotional interdependence: the key to studying extrinsic emotion regulation

**DOI:** 10.1186/s41155-022-00238-8

**Published:** 2022-12-02

**Authors:** Ana Kinkead, Christian Salas Riquelme

**Affiliations:** 1 Universidad Autónoma de Chile, Av. Pedro de Valdivia 425, Providencia, Santiago, Chile; 2 Faculty of Psychology, Universidad Diego Portales, Santiago, Chile

**Correction: Psicologia: Reflexão e Crítica 35, 35 (2022)**

**https://doi.org/10.1186/s41155-022-00237-9**

Following publication of this article (Kinkead & Riquelme, 2022), the “**BLINDED**” in the citation (Jitaru, 2020; **BLINDED**) should be changed to the **Kinkead et al., 2021**.

The below reference also needs to be added:

Kinkead, A., Sanduvete-Chaves, S., Chacón-Moscoso, S., Salas, C. (2021). Couples’ Extrinsic Emotion Regulation Questionnaire: Psychometric Validation in a Chilean Population. PLoS ONE2. 16(6): e0252329. https://doi.org/10.1371/journal.pone.0252329

The original article (Kinkead & Riquelme, 2022) was updated.
